# Hydrogen Atom Transfer from HOO^.^ to *ortho*‐Quinones Explains the Antioxidant Activity of Polydopamine

**DOI:** 10.1002/anie.202101033

**Published:** 2021-06-04

**Authors:** Yafang Guo, Andrea Baschieri, Fabio Mollica, Luca Valgimigli, Jakub Cedrowski, Grzegorz Litwinienko, Riccardo Amorati

**Affiliations:** ^1^ Department of Chemistry “G. Ciamician” University of Bologna Via S. Giacomo 11 40126 Bologna Italy; ^2^ Faculty of Chemistry University of Warsaw Pasteura 1 02-093 Warsaw Poland

**Keywords:** antioxidants, peroxyl radicals, polydopamine, quinone, radical reactions

## Abstract

Melanins are stable and non‐toxic biomaterials with a great potential as chemopreventive agents for diseases connected with oxidative stress, but the mechanism of their antioxidant action is unclear. Herein, we show that polydopamine (**PDA**), a well‐known synthetic melanin, becomes an excellent trap for alkylperoxyl radicals (ROO^.^, typically formed during autoxidation of lipid substrates) in the presence of hydroperoxyl radicals (HOO^.^). The key reaction explaining this peculiar antioxidant activity is the reduction of the *ortho*‐quinone moieties present in **PDA** by the reaction with HOO^.^. This reaction occurs via a H‐atom transfer mechanism, as demonstrated by the large kinetic solvent effect of the reaction of a model quinone (3,5‐di‐tert‐butyl‐1,2‐benzoquinone) with HOO^.^ (*k*=1.5×10^7^ and 1.1×10^5^ M^−1^ s^−1^ in PhCl and MeCN). The chemistry disclosed herein is an important step to rationalize the redox‐mediated bioactivity of melanins and of quinones.

## Graphical abstract

Polydopamine, a melanin analogue, is an excellent trap for alkylperoxyl radicals (ROO^.^, typically formed during autoxidation of lipid substrates) only in the presence of hydroperoxyl radicals (HOO^.^). Activation of the *ortho*‐quinone moieties by HOO^.^, and formation of exposed semiquinones are proposed as the reason of the peculiar antioxidant activity.

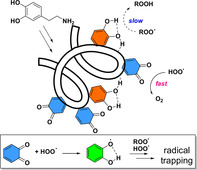

Melanins are a family of intensely colored polymers derived from oxidative polymerization of phenols such as tyrosine, DOPA and dopamine.[^1^, ^2^] Although the structure of melanins is not fully clarified, there is evidence that it consists of reduced (catechol) and oxidized (*ortho*‐quinone) units (see Scheme 1 A).[^3^, ^4^] Beside their important role in living organisms,[^5^, ^6^, ^7^] melanins are currently being investigated for many applications, including energy storage, biocompatible adhesion or coating systems, drug delivery, and skin protection.[^5^, ^6^, ^8^] Melanins have been proposed to possess a “multi‐antioxidative” activity,[^4^, ^5^, ^8^, ^9^] that has been related, for instance, to their anti‐inflammatory,^[10]^ wound regeneration^[11]^ and anti‐ischemic activity.^[12]^ Natural eumelanin incorporating large amounts of 5,6‐dihydroxyindole‐2‐carboxylic acid, DHICA (see Scheme 1 A), has been reported to have the strongest capability to quench radicals, as compared to synthetic melanins obtained by dopamine polymerization.^[5]^ Polydopamine traps HO^.^ radicals and has superoxide dismutase (SOD)‐like activity (see Scheme 1 B) presumably involving the stable radicals “hosted” inside melanins.[^8^, ^9^] Despite the number of publications reporting the antioxidant activity of melanins is steadily increasing, the mechanism of radical trapping remains unclear. Most mechanistic studies have been performed by using stable radicals such as DPPH^.^ and ABTS^.+^ (Scheme 1 B).[^7^, ^9^] While these pioneering works have triggered the interest on melanins’ redox properties, these artificial radicals have limited similarity to transient alkylperoxyl radicals (ROO^.^) (Scheme 1 C).^[13]^ The structure of melanins at the molecular level largely depends on the nature of the phenolic monomer,[^7^, ^14^] on the polymerization conditions[^14^, ^15^] and on post‐synthetic functionalization.^[12]^ Despite the efforts in this direction,^[16]^ there is currently no accepted simplified model for the redox properties of melanins. A good starting point to face this problem would be having a clear understanding of the radical trapping behavior of the basic structural units that are present in all melanins, namely the 1,2‐dihydroxy benzene (catechol) and the 1,2‐benzoquinone moieties.[^17^, ^18^, ^19^]

**Scheme 1:**
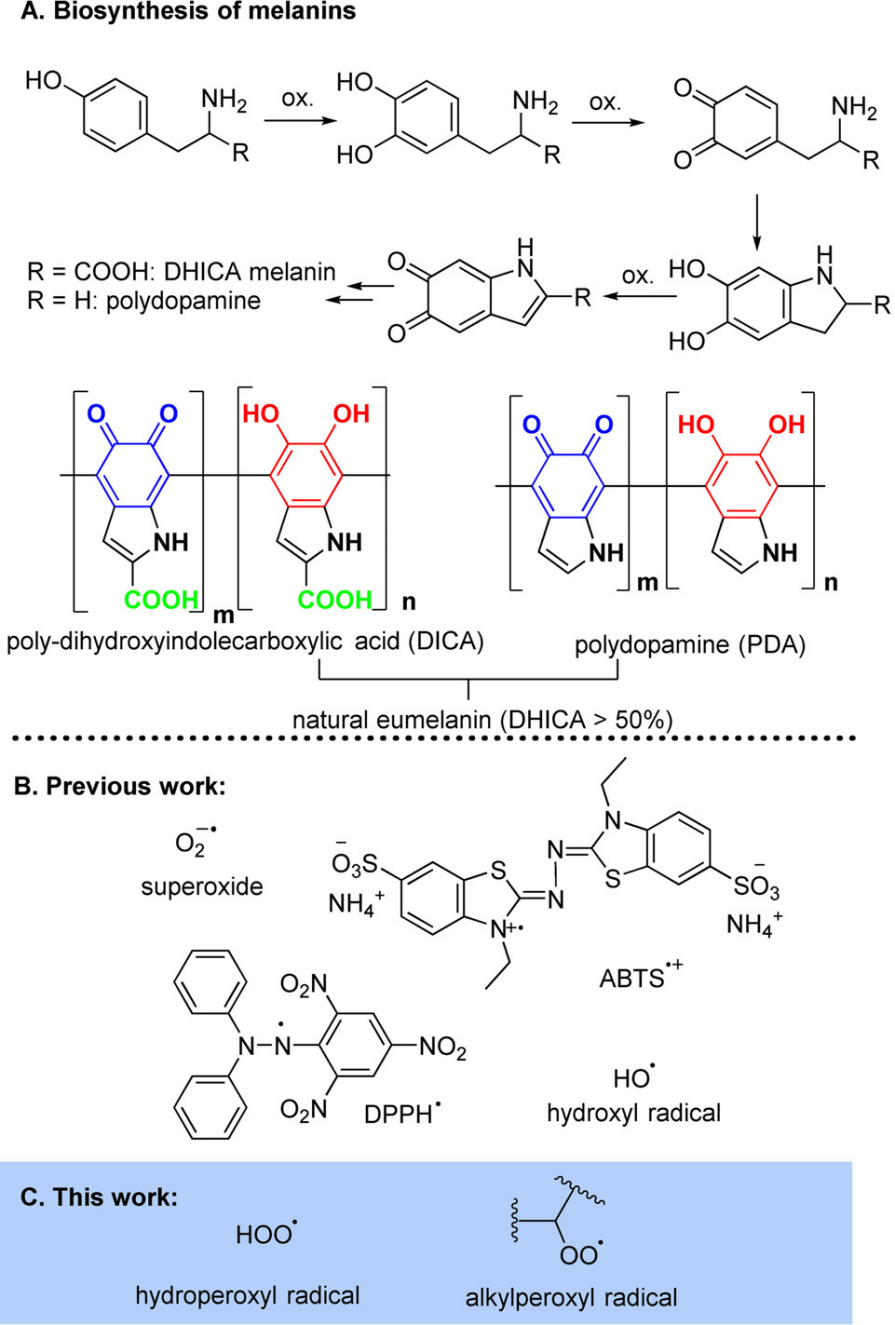
(A) (Bio)synthetic pathway leading to the formation of melanins; (B,C) radical used to investigate the antioxidant activity of melanins.

Herein we report the results of our studies of controlled radical chain oxidations carried on by mixed alkylperoxyl (ROO^.^) and hydroperoxyl (HOO^.^) radicals[^20^, ^21^, ^22^, ^23^] to understand the reactivity and the mechanisms of antioxidant action of polydopamine (**PDA**, Scheme 1 A) and of two model *ortho* (**1**) and *para* (**2**) quinones (Scheme 2).

**Scheme 2:**
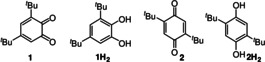
Model compounds investigated in this study.

The chain‐breaking antioxidant activity of **1** and **2** was evaluated by measuring, at 30 °C and in a low‐polarity solvent (chlorobenzene), the rate of O_2_ consumption during the azo‐bis(isobutyronitrile) (AIBN) initiated oxidation of styrene, a reference organic substrate whose autoxidation at low temperature is propagated by ROO^.^.[^24^, ^25^] 1,4‐Cyclohexadiene (CHD) was used as oxidizable co‐substrate to produce HOO^.^ [(Scheme 3, Eqs. (8) and (9)].[^20^, ^21^, ^22^, ^23^] As expected,[^17^, ^18^, ^19^] **1** and **2** had no effect on the peroxidation of styrene alone (Figure 1 A). When CHD was added to styrene, both quinones suppressed the peroxidation, as shown in Figures 1 A and S1.

**Figure 1:**
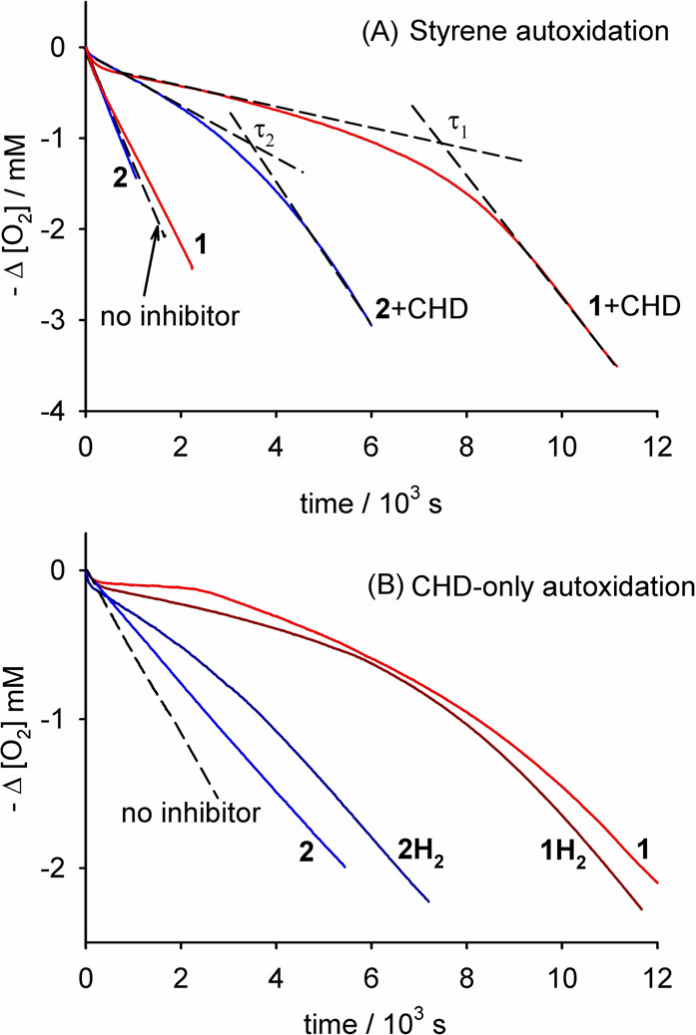
A) O_2_ consumption during the autoxidation of styrene in chlorobenzene at 30 °C initiated with 25 mM AIBN in the absence of inhibitors (dashed line), and in the presence of quinones **1** and **2** (5 μM) with or without 0.023 M CHD. B) O_2_ consumption during the autoxidation of CHD (0.23 M) initiated by 25 mM AIBN at 30 °C in chlorobenzene with no antioxidants (dashed line), or with added 40 μM of **2** or **2H_2_**, and with 5 μM **1H_2_** or **1**.

**Scheme 3:**
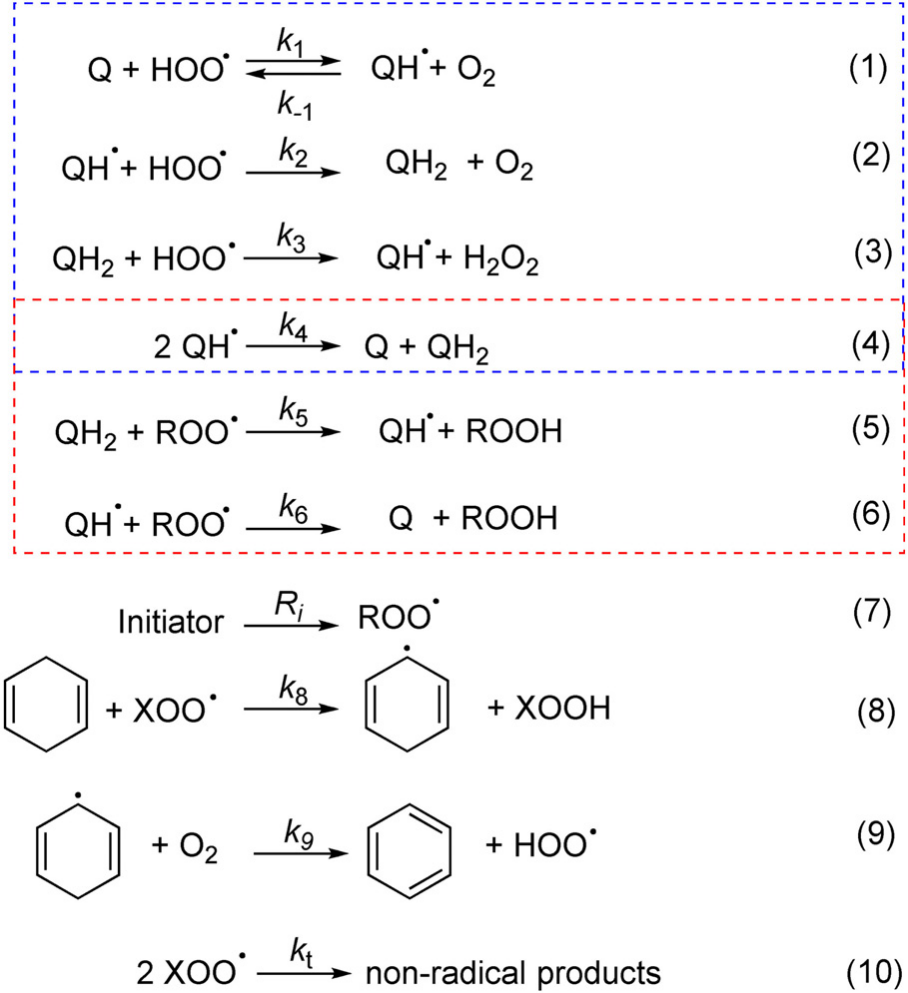
Key reactions explaining the antioxidant activity of *ortho* and *para*‐quinones, **Q**, in the presence of 1,4‐cyclohexadiene (X=H or R).

Control experiments (styrene containing CHD but without quinones) indicated neither inhibition nor retardation, thus the strong inhibition observed for styrene/CHD/quinone can be explained by the reactions reported in Scheme 3. Comparing the kinetic traces in Figure 1, we should notice that **1** generates longer induction period than **2** used at the same concentration (see τ_1_ and τ_2_) and, additionally, the rate of O_2_ uptake during the inhibition period is smaller for **1** than for **2**, demonstrating a superior antioxidant activity of the *ortho*‐isomer. To better clarify the mechanism proposed in Scheme 3, we completely replaced styrene with CHD. Figure 1 B presents the kinetic traces recorded for the system, with the autoxidation propagated exclusively by HOO^.^ radicals.^[22]^ Quinone **1** almost completely stopped the autoxidation of CHD (i.e. it trapped HOO^.^ with high efficiency), while **2** was less effective even if used at much higher concentration. We also employed the reduced species, catechol/ hydroquinone, **1H_2_** and **2H_2_** to test their behavior during autoxidation of CHD. Results, reported in Figure 1 B, indicated a smaller and a higher HOO^.^ trapping ability than the corresponding quinones for **1H_2_** and **2H_2_** respectively.^[26]^

The use of CHD as the only oxidizable substrate simplifies the analysis of autoxidation kinetics, propagated only by HOO^.^ [X=H in Eqs. (8) and (10)], since reactions (5) and (6) become unimportant. Having clarified that the antioxidant activity of *ortho*‐benzoquinone **1** stems from its reaction with HOO^.^ [Eq. (1)], in the initial stages of autoxidation when the inhibitor is **1**, before a substantial accumulation of reduced **1H_2_** [Eq. (2)], reaction (3) also becomes unimportant. Therefore, the rate constant for reaction (1) can be determined in first approximation by using Equation 11, which relates the rates of the inhibited and non‐inhibited autoxidation (*R*_inh_ and *R*_0_, respectively) to the rate constant for the reaction of the antioxidant with the chain‐carrying radicals and the stoichiometry of radical trapping (*n*).^[25]^

(11) $$\left(R{}_{0}/R{}_{\mathrm{in}}\right)-\left(R{}_{\mathrm{in}}/R{}_{0}\right)=n\;k{}_{1}\;\left[\mathrm{AH}\right]/\;\left(2\;k{}_{t}\;R{}_{i}\right){}^{1/2}$$

The rate of initiation (*R*_i_) was determined experimentally as 3.1×10^−9^ Ms^−1^, while 2*k*_t_ for CHD in chlorobenzene is 1.2×10^9^ M^−1^ s^−1^.^[22]^ With the assumptions that the rate of the back reaction *k*_−1_ is negligible and that *n=*2, *k*_1_ was obtained as (1.4±0.2)×10^7^ M^−1^ s^−1^. Numerical modeling of O_2_ consumption traces of CHD and styrene autoxidations, inhibited either by **1** or **1H_2_**, was then performed by using the full array of kinetic equations and the COPASI kinetic simulation software (see Supporting Information)[^27^, ^28^] as illustrated in Figure 2 A. The optimized values of *k*_1_ and *k*_3_ obtained with this procedure were 1.5×10^7^ and 1.5×10^6^ M^−1^ s^−1^, respectively, while for *k*_−1_ an upper limit of 65 M^−1^ s^−1^ was determined. Hence our results show that, counterintuitively, the oxidized quinone **1** is a far better antioxidant than catechol **1H_2_**, but only in the presence of HOO^.^. Results also show that a substantial quantity of catechol **1H_2_** is formed from **1** (Figure 2 A). This finding is again counterintuitive, as it means that the antioxidant is being reduced, albeit transiently, during the inhibition of CHD autoxidation. Formation of **1H_2_** was confirmed by ESI‐MS analysis performed on the reaction mixture containing **1**, CHD and AIBN (Figure 2 B,C). Quinone **1** was visible only in positive ion mode mainly as **1**+Na^+^ peak, while catechol **1H_2_** could be detected in negative ion mode as **1H^−^**. The same result was obtained by GC‐MS, after derivatization of the sample with trimethylsilyl‐N,N‐dimethylcarbamate (TMSDMC) as silylating agent to protect **1H_2_** from decomposition during the analysis (see Figures S5–S7).

**Figure 2:**
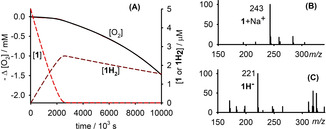
(A) Numerical fitting of the O_2_ consumption traces during the autoxidation of CHD initiated by AIBN in PhCl at 30 °C inhibited by **1**. Experimental results for oxygen consumption (black, solid line) and the simulated results (red line), perfectly overlapping each other. The transient concentrations of the quinone or hydroquinone species obtained during the simulations are reported. (B,C) ESI‐MS spectra showing **1H_2_** formation during the reaction of **1** (0.1 mM) with CHD (0.23 M) and AIBN (50 mM) in MeCN at 30 °C; after 1 hour of reaction in positive (B) and negative (C) ion mode.

Analysis of the kinetic solvent effect (KSE) offered further insight in the reaction of **1** with HOO^.^. In acetonitrile, the inhibiting effect of **1** during the autoxidation of CHD was weaker than in PhCl (see Figures S8 and S9). Numerical fitting provided *k*_1_=1.1×10^5^ M^−1^ s^−1^, that is 180‐folds smaller than in PhCl. This type of KSE is well established for antioxidants whose mechanism relies on a rate‐limiting formal H‐atom transfer, which is hampered by occurrence of H‐bonding to the solvent.^[13]^ Normally, when the antioxidant is a phenol transferring the H‐atom to a peroxyl radical, phenol itself is the H‐bond donor (HBD), complexing to the solvent.[^13^, ^29^, ^30^] Here, instead, the role is reverted and the HOO^.^ radicals act as the HBD. From the observed magnitude of this KSE, the ability of HOO^.^ as H‐bond donor, H‐bond acidity parameter α_2_^H^, can be calculated^[29]^ as 0.78, in reasonable agreement with previous estimates.[^20^, ^31^] KSE is a proof that the reaction of **1** with HOO^.^ involves a H‐atom transfer (HAT) and that this process starts with formation of a pre‐reaction H‐bond complex competing with the solvent (Scheme 4).

**Scheme 4:**
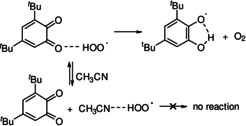
Kinetic solvent effect for H‐atom donation from HOO^.^ to quinones.

Quantitative understanding of the reactions of these model structures could then be transferred to rationalize the behavior of PDA as a representative member of melanin biopolymers. Polydopamine (**PDA**) nanoparticles were synthetized by the oxidative polymerization of dopamine in alkaline water/ethanol solution and were accurately purified by repeated centrifugation cycles (characterization by DLS, TEM and FT‐IR is reported in Figure 3, S10–S12).[^32^, ^33^] The nanoparticles were concentrated, dispersed in acetonitrile and then used as inhibitor in autoxidation studies. As shown in Figure 3 C, **PDA** itself had a modest antioxidant activity toward styrene autoxidation (solvent MeCN), presumably due to the low concentration of catechol moieties, or their engagement in strong intramolecular H‐bonds.^[34]^ Actually, on further extending the purification cycles, **PDA** showed even lower inhibition of autoxidation (Figure S13), suggesting that some catechol moieties could be physically trapped into the nanoparticle matrix, instead of being covalently bound, or could exist as small (extractable) oligomers or covalent adducts, such as the pyranoacridinetrione recently proposed.^[35]^ Interestingly, after the addition of CHD the antioxidant activity of **PDA** became very prominent (Figure 3 C trace d). Under the same conditions, CHD had a negligible effect on **1H_2_** (Figure 3 D traces d and e), while it greatly increased the activity of **1** (traces c and f). The modest reactivity of **PDA** toward ROO^.^ can be explained by considering that the catechol moieties are strongly bonded to H‐bond acceptors, such as carbonyl groups, present into the polymer (Figure 3 E).^[36]^ Not H‐bonded catechols which would be more reactive toward radicals than H‐bonded ones,^[13]^ are most probably oxidized during the preparation of **PDA**. Instead, upon reaction with HOO^.^, *ortho*‐quinones can form “exposed” semiquinones and catechol groups able to trap both HOO^.^ and ROO^.^.

**Figure 3:**
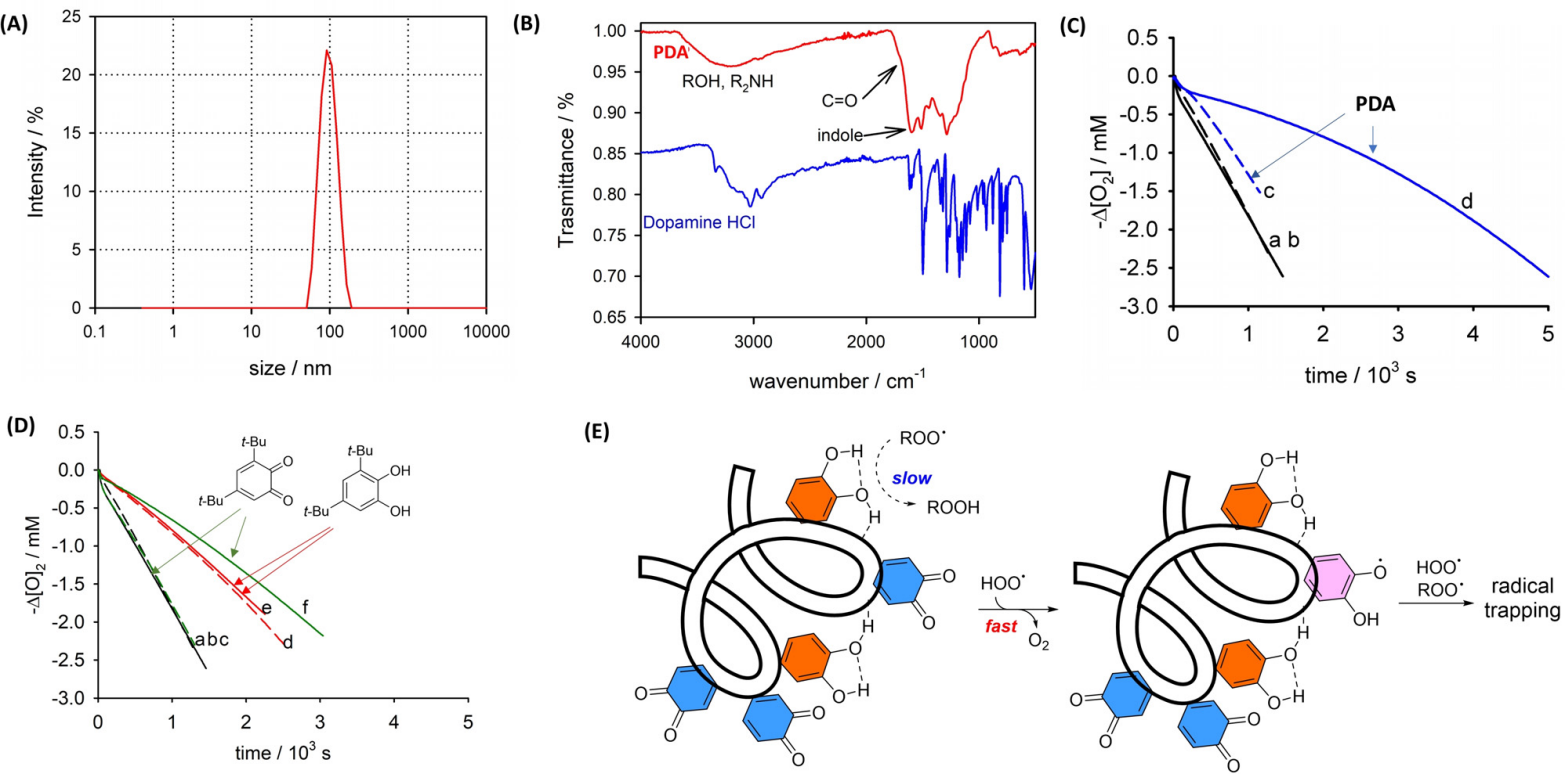
(A) Dynamic light scattering of **PDA** nanoparticles in water. (B) ATR‐FTIR spectrum of dried **PDA**. (C) O_2_ consumption recorded during the styrene (2.1 M) autoxidation initiated by AIBN (25 mM) in MeCN without inhibitors (a) and in the presence of: (b) CHD; (c) **PDA** (25 μg mL^−1^); (d) **PDA** (25 μg mL^−1^) + CHD. (D) Same conditions as C, without inhibitors (a) and in the presence of: (b) CHD; (c) **1** (5 μM); (d) **1H_2_** (5 μM); (e) **1H_2_** (5 μM) + CHD; (f) **1** (5 μM) + CHD. In all cases, [CHD]=23 mM. (E) Catechol and quinone units in the polydopamine polymer are unreactive toward alkylperoxyl radicals (ROO^.^) but upon the reaction with HOO^.^ the quinones are converted to *ortho*‐semiquinone radicals with enhanced ability to trap both species, HOO^.^ or ROO^.^.

To rationalize the antioxidant activity of **PDA**, the driving force for the H‐atom transfer reaction of HOO^.^ to **1**, **2** and to the quinones relevant to the chemistry of **PDA** (**3**–**6**) was calculated (see Figure 4, S14–S17). The reaction of HOO^.^ with **1** is much more exothermic than with **2**, in agreement with the results from CHD autoxidation studies (see Figure 1) and with previous reports.[^37^, ^38^] Dopaminochrome (**4**) (transiently formed during **PDA** synthesis)^[7]^ doesn't react as efficiently with HOO^.^ as dopaminoquinone (**3**) and indole‐5,6‐quinone (**5**). Interestingly, the indole‐5,6‐quinone‐5,6‐dihydroxyindole dimer (**6**), proposed as building block of **PDA**, shows a high reactivity toward HOO^.^, fully consistently with experimental results. Moreover, o*rtho*‐semiquinones are stabilized by a strong intramolecular H‐bond and are therefore generally unreactive toward O_2_.^[37]^ These calculations further support our proposed mechanism to explain the antioxidant behavior of **PDA**. Regarding the semiquinone‐type radicals naturally hosted in **PDA** (and in other melanins), contributing to the persistent EPR signal,[^4^, ^5^] their rapid reduction to catechols by HOO^.^ [Eq. (2)]—a reaction expected to be diffusion controlled from our numerical fittings—is also likely to have a role in the enhanced antioxidant behavior in the presence of HOO^.^, although its actual contribution might deserve further investigation.

**Figure 4:**
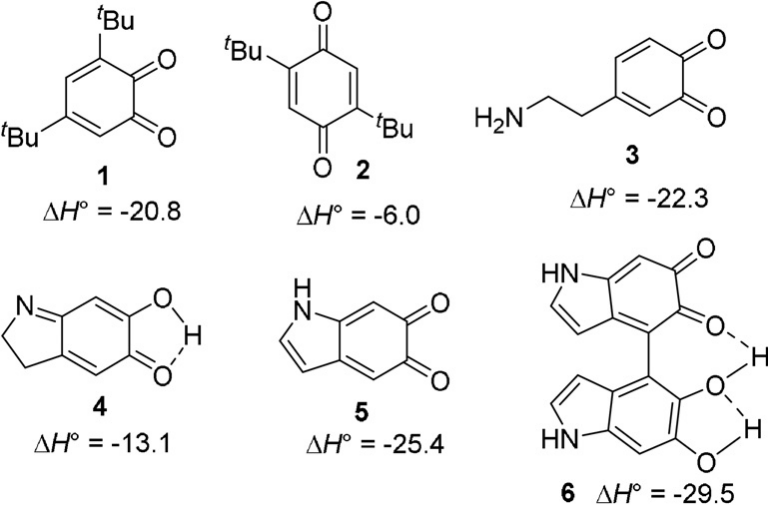
Calculated enthalpy variation for the reaction of H‐atom transfer from HOO^.^ to quinones [reaction (1)] in kcal mol^−1^. The most stable tautomers of the quinones and of the semiquinone radicals are considered (level: CBS‐QB3 or B3LYP/6‐311++g(d,p) for **6**, gas phase).

The simultaneous presence of alkylperoxyl and hydroperoxyl radicals is a common feature in biological systems.^[39]^ Alkylperoxyl radicals are responsible for lipid peroxidation of unsaturated membranes,^[24]^ while the HOO^.^ radicals are constantly produced by protonation of superoxide leaking from the mitochondrial respiratory chain and by the autoxidation of alcohols, aliphatic amines and alkenes.[^21^, ^23^, ^40^] The chemistry disclosed herein is an important step to rationalize the redox‐mediated bioactivity of **PDA** and the prominent antioxidant chemistry of *ortho* and *para*‐quinones. With the caution imposed by their distinctive chemical diversity,^[14]^ it could also serve as a rational basis to understand the properties of other melanins and it might be implemented for the rational design of novel antioxidant biomaterials that can be selectively activated by hydroperoxyl radicals.

## Conflict of interest

The authors declare no conflict of interest.

## Supporting information

As a service to our authors and readers, this journal provides supporting information supplied by the authors. Such materials are peer reviewed and may be re‐organized for online delivery, but are not copy‐edited or typeset. Technical support issues arising from supporting information (other than missing files) should be addressed to the authors.

Supplementary
