# Supplementary material for: Hydrogen Atom Transfer from HOO. to ortho‐Quinones Explains the Antioxidant Activity of Polydopamine
Source: Angew Chem Int Ed Engl. 2021 Jun 4;60(28):15220–4. doi: 10.1002/anie.202101033 (PMC8362028; doi:10.1002/anie.202101033)
Supplement: Supplementary file 1 — Supplementary [file ANIE-60-15220-s001.pdf]

Angewandte Chemie
Eine Zeitschrift der Gesellschaft Deutscher Chemiker

Supporting Information

Hydrogen Atom Transfer from HOO$^{\cdot}$ to *ortho*-Quinones Explains the Antioxidant Activity of Polydopamine

*Yafang Guo, Andrea Baschieri, Fabio Mollica, Luca Valgimigli, Jakub Cedrowski, Grzegorz Litwinienko, and Riccardo Amorati**

anie_202101033_sm_miscellaneous_information.pdf

**Content**	**page**
Experimental section	2-3
Figures S1 and S2.	4
Kinetic analysis by COPASI software	5-6
Kinetic scheme used for numerical fitting	7-9
Product analysis GC-MS	10-12
Autoxidation in MeCN	13
Characterization of PDNPs	14-16
Inhibition of the autoxidation of styrene/CHD by **PDA**	17
Results from theoretical calculation	18
References	23

Experimental section

Materials. All solvents (PhCl, MeCN, ethanol) were of the highest grade commercially available (≥99.9% HPLC grade) and used as received. Deionized water was Millipore grade. Styrene and 1,4-cyclohexadiene (CHD, 97%, stabilized by hydroquinone) was percolated on alumina and silica before each experiment in order to remove traces of stabilizer. The initiator, 2,2′-azobis(2-methylpropionitrile) (AIBN), was recrystallized from methanol before use. Dopamine hydrochloride, 3,5-di-tert-butyl-1,2-benzoquinone **1**, 2,5-di-tert-butyl-1,4-benzoquinone **2**, 3,5-di-*tert*-butyl-catechol **1H$_2$**, 2,5-di-*tert*-butyl-1,4-hydroquinone **2H$_2$** were from Sigma-Aldrich and were used as received**.**

Synthesis of polydopamine nanoparticles. Ethanol (3 mL) was mixed with deionized water (7 mL) and dopamine hydrochloride (25 mg) under mild magnetic stirring at 50°C for 30 min.[1,2] A solution of ammonium hydroxide (NH_3 in H_2O 28% w/w, 100 μL) was cooled and then injected into the above mixed solution, and let react for 24 h. The material was cleaned by five consecutive centrifugation (15000g for 10 min) and washing cycles (Batch 1). A small aliquot was further purified by two centrifugation cycles (Batch 2) and used as comparison for autoxidation studies (see Figure S13) to check the purification efficacy. The concentration of the nanoparticle suspension (Batch 1) was 0.5 mg/mL, as determined by weighing the mass after drying. The nanoparticles were characterized by dynamic light scattering (DLS), transmission electron microscopy (TEM) and attenuated total reflection infrared spectroscopy (ATR FT-IR) as described in Figures S10-S12. The nanoparticles were then concentrated by centrifugation (15000 g), re-dispersed in acetonitrile (final concentration 0.5 mg/mL) before being used as inhibitors of the styrene autoxidation.

Measurements of the antioxidant activity. Oxygen consumption was measured in a two-channel gas uptake apparatus, immersed in a thermostatic bath, based on Validyne DP15 pressure transducer,[3,4,5] suitable also for the study of nanomaterials. The rate of initiation (R_i) was calculated from preliminary set of experiments from the length of the inhibition period, τ, using 2,2,5,7,8-

pentamethyl-6-chromanol as a reference antioxidant during autoxidation of styrene. Numerical fitting of the experimental O_2 consumption traces were performed by using the kinetic simulation software COPASI, freely available on the Internet.[6] The reaction scheme used to simulate the experimental data is reported in the Supporting Information.

ESI-MS Measurements. Mass spectra were obtained by direct infusion with a microsyringe pump (15 μL/min) into a Micromass ZMD ESIMS spectrometer using the following instrumental settings: positive ions; desolvation gas (N_2), 250 L/h; cone gas (skimmer), 22 L/h; desolvation temperature, 100 °C; capillary voltage, 3.0 kV; cone voltage, 10-40 V; hexapole extractor, 3 V; RF lens, 0.3 V.

Theoretical calculations.

Geometry optimization and enthalpies were calculated using the CBS-QB3 complete basis set method[7] as implemented in the Gaussian 16 suite of programs.[8] The ΔH of reaction of quinones and HOO• were obtained by calculating the relevant enthalpies-corrected energies for the following reaction:

BDE(QH•) $\quad$ QH• → Q + H• $\quad$ (1S)

BDE(HOO•) $\quad$ HOO• → $^3O_2$ + H• $\quad$ (2S)

ΔH° = BDE(HOO•) – BDE(QH•)

When different tautomeric Q or QH• were possible, only the most stable ones were considered (see Table S5). In the case of the dimer, the geometry was optimized at the B3LYP/6-311+G(d,p) level and stationary points were confirmed by checking the absence of imaginary frequencies. The BDE(QH•) was then obtained by using the isodesmic approach, by using phenol as reference (BDE PhO-H = 86.7 kcal mol^{-1}).[9]

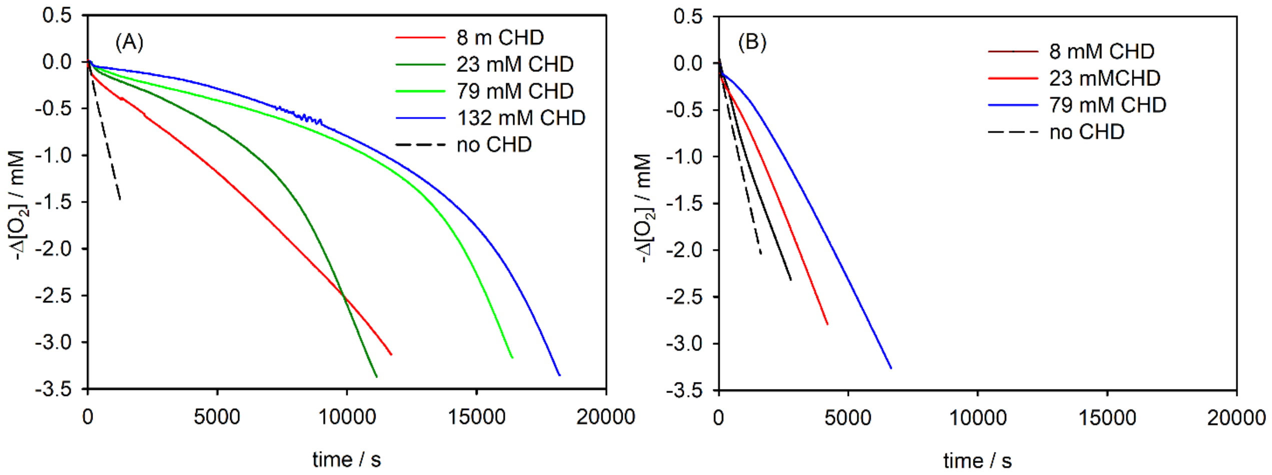

Figure S1. O_2 consumption during the autoxidation of styrene in chlorobenzene at 30 °C initiated with 25 mM AIBN in the absence of inhibitors in the presence of 5 μM of quinone **1** (A) or **2** (B) and increasing amounts of CHD.

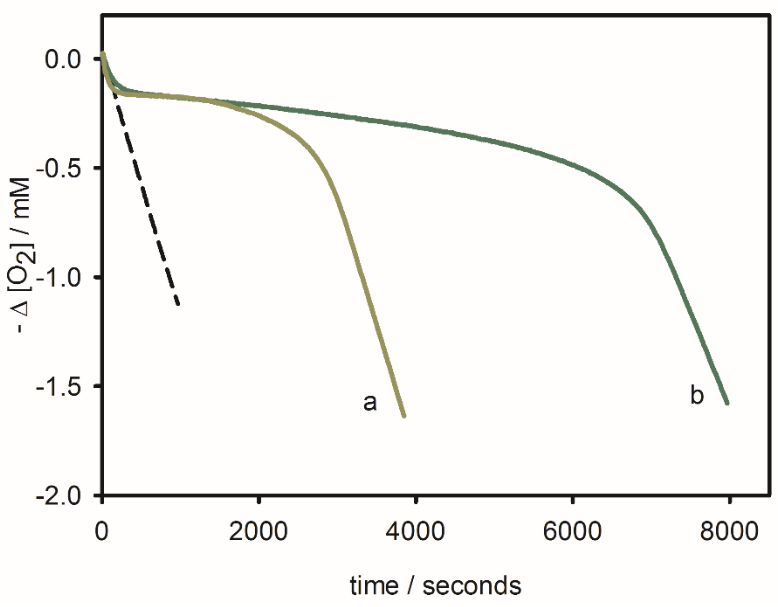

Figure S2. O_2 consumption measured during the autoxidation of styrene (4.3 M) in PhCl initiated by AIBN (0.25 mM) at 30 °C in the absence of antioxidants (dashed line) or in the presence of (a) **$2H_2$**, (b) **$1H_2$** (both 10 μM).

Kinetic analysis by COPASI software

The numerical modeling of some representative O_2 consumption traces were performed by using a kinetic simulation software (Copasi).[6] This software was used previously in other studies by us and by other research groups to analyze the autoxidation kinetics.[10,11] We used the kinetic equations reported in Scheme 3 in addition to those describing the autoxidation of CHD or styrene and all the termination reactions. The propagation (k_p) and termination ($2k_t$) rate constants in chlorobenzene at 30 °C are, respectively, 1400 and 1.2×10^9 $M^{-1}s^{-1}$ for CHD[12] (for CHD, $k_p \equiv k_8$), and 41 and 4.2×10^7 $M^{-1}s^{-1}$ for styrene.[3] The rate constant k_4 for disproportionation of **1H•** was assumed to be the same as for the 3,6-di-*tert*-butyl-2-hydroxyphenoxyl radical in benzene at room temperature (8.0×10^5 $M^{-1}s^{-1}$)[13] because of similar steric crowding around the phenoxyl oxygen. The rate constants k_2 and k_9, i.e. for radical-radical cross terminations, were assumed to be 2.0×10^9 $M^{-1}s^{-1}$, and the rate constant k_{11} was let free to vary in the range (1-50) $\times10^8$ $M^{-1}s^{-1}$.[14] With these approximations, we could reproduce with good accuracy the experimental O_2 consumption traces as illustrated in Figure 2A. In the case of **1** in CHD, its concentration was adjusted to account for its slightly shorter inhibition period (by about 20%) typically observed, probably due to a side reaction not included into the reaction scheme (for our attempts aimed at clarifying this point)[15]. Different sets of k_1 and k_{-1} values provided a good fitting of the O_2 consumption traces, indicating that these constants cannot be determined independently in a single experiment. However, we noticed that for CHD inhibited by **1**, the rate of O_2 consumption is most sensitive to the **1** + HOO• reaction (i.e. to k_1), whereas for styrene inhibited by **1H₂**, the *length* of the induction period depends on **1H•** + O_2 reaction (i.e on k_{-1}). Therefore, we adopted the graphical approach shown in Figure S3, with k_1 as a function of k_{-1} for CHD autoxidation inhibited by **1** and for styrene autoxidation inhibited by **$1H_2$** (Figure 3S). The crossing point of these plots is the best estimate for the two rate constants. The values of k_1, k_{-1} and k_5 obtained with this procedure were 1.5×10^7 $M^{-1}s^{-1}$, 65 $M^{-1}s^{-1}$and 1.5×10^6 $M^{-1}s^{-1}$, respectively. Interestingly, the k_1 value obtained by numerical fitting is in excellent agreement with that found by using equation 11, the reason being that the k_{-1} value is small enough to make the slope of O_2 consumption during CHD

autoxidation sensitive only to the k_1 value. The rate constant for the reaction of **1H₂** with HOO• (k_3 = 1.5 ×10^6 $M^{-1}s^{-1}$) is very similar to that previously recorded for the same catechol with ROO• (1.1 ×10^6 $M^{-1}s^{-1}$ at 303K in chlorobenzene)[16] confirming that HOO• and ROO• have similar oxidizing reactivity.

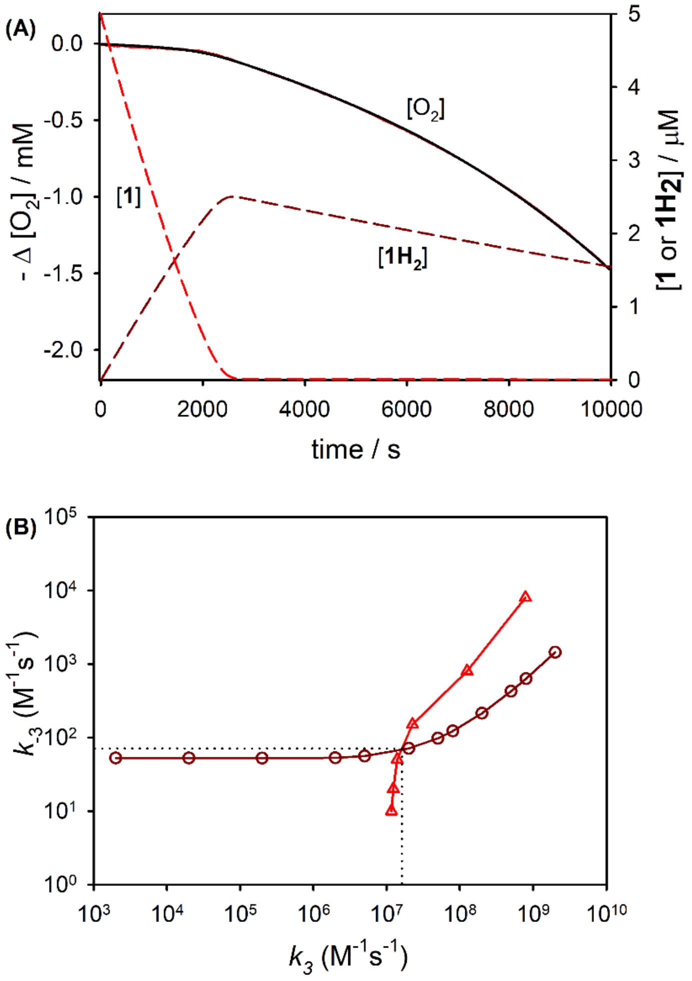

Figure S3. A) Numerical fitting of the O_2 consumption traces during the autoxidation of CHD initiated by AIBN in PhCl at 30 °C inhibited by **1**. Experimental results for oxygen consumption (black, solid line) and the simulated results (red line) perfectly overlap each other. The transient concentrations of the quinone or hydroquinone species obtained during the simulations are reported. B) Values of k_{-1} as a function of k_1 (logarithmic scale) obtained by numerical fitting of the rates of CHD autoxidations inhibited by **1** (Δ) or of styrene autoxidation inhibited by **1H₂** (○).

Kinetic scheme used for numerical fitting

Table S1. Species considered in numerical fitting of the autoxidation of 1,4-cyclohexadiene (CHD) initiated by AIBN at 30 °C.

**chemical name**	**symbol used in equations**	**Initial concentration**
I	Initiator	not relevant. Radical initiation is determined directly by Ri.
HOO	HOO•	0
R	cylohexadienyl alkyl radical	0
O2	dissolved O_2	1.8e-3
O2g	O_2 in the headspace	1 M [a]
RH	1,4-cyclohexadiene	depending on experiment
HOOH	H_2O_2	0
QH2	1,4-di-tert-butyl catechol	depending on experiment
QH	radical from 1,4-di-tert-butyl catechol	0
Q	1,4-di-tert-butyl ortho quinone	depending on experiment
P	non radical product	0

a) The concentration of O_2 in the headspace is set to an arbitrary high value to ensure that the dissolved O_2 concentration remains constant. This approximation holds because during the experiment only a small fraction of O_2 in the system is consumed. In a typical autoxidation experiment, less than 30% of total O_2 is consumed.

Table S2. Kinetic scheme considered in numerical fitting of the autoxidation of 1,4-cyclohexadiene (CHD) initiated by AIBN at 30 °C in chlorobenene.

N	Reaction **Equations**	Rate **Constants** ($M^{-1}S^{-1}$) or **Fluxes** (Ms^{-1})	**Notes and references**
**1**	I -> HOO	v = 2.5e-009 [a]	Initiation rate (Ri), depends on experiment
**2**	R + O2 -> HOO + B	k = 5e+009	B. Maillard, K. U. Ingold, J. C. Scaiano J. Am. Chem. Soc. 1983, 105, 5095-5099
**3**	HOO + RH -> HOOH + R	k = 1400	Howard, J. A.; Ingold, K. U. Can. J. Chem. 1965, 43, 2729–2736
**4**	HOO + HOO -> O2 + HOOH	k = 6.3000e+008	Howard, J. A.; Ingold, K. U. Can. J. Chem. 1965, 43, 2729–2736
**5**	QH2 + HOO -> QH + HOOH	k = fit	initial guess: k=1e6
**6**	QH + HOO -> Q + HOOH	k = 2e9	assumed
**7**	QH + HOO -> QH2 + O2	k = 2e9	assumed
**8**	O2g = O2	k1 = 1.9e+6<br>k2 = 1e+009	assumed. Equilibrium constant from O2 solubility in benzene.
**9**	QH+QH->Q+QH2	k = 8e5	Tumanskii, B. L.; Solodovnikov, S. P.; Prokof'ev, A. I.; Bubnov, N. N.; Kabachnik,

			M. I.; Ivz. Akad. Nauk SSSR Ser. Khim. 1977, 1309 [from Landolt–Börnstein]
**10**	Q+HOO = QH+O2	k1 = fit<br>k2 = fit	
**11**	QH + HOO -> P	k = fit	initial guess: k=1e8 E. T. Denisov, I. V. Khudyakov Chem. Rev. 1987, 87, 1313-1357

a) constant flux.

Table S3. Species considered in numerical fitting of the autoxidation of styrene initiated by AIBN at 30 °C in chlorobenzene.

**chemical name**	**symbol used in equations**	**Initial concentration**
I	Initiator	not relevant. Radical initiation is determined directly by Ri.
ROO	styryl peroxyl radicals	0
R	styryl alkyl radical	0
O2	dissolved O_2	1.8e-3
O2g	O_2 in the headspace	1 M [a]
RH	styrene	depending on experiment
ROOH	styrene polyperoxide or hydroperoxide	0
QH2	1,4-di-tert-butyl catechol	depending on experiment
QH	radical from 1,4-di-tert-butyl catechol	0
Q	1,4-di-tert-butyl ortho quinone	depending on experiment
HOO	HOO•	0
HOOH	H_2O_2	0
P	non radical product	0

a) The concentration of O_2 in the headspace is set to an arbitrary high value to ensure that the dissolved O_2 concentration remains constant. This approximation holds because during the experiment only a small fraction of O_2 in the system is consumed. In a typical autoxidation experiment, less than 30% of total O_2 is consumed.

Table S4. Kinetic scheme of the autoxidation of styrene initiated by AIBN at 30 °C in chlorobenzene.

N	Reaction Equations	Rate **Constants** ($M^{-1}s^{-1}$) or **Fluxes** (Ms^{-1})	Notes and references
**1**	I -> R	v = 5e-009	Initiation rate (Ri), depends on experiment
**2**	R + O2 -> ROO	k = 5e+009	B. Maillard, K. U. Ingold, J. C. Scaiano J. Am. Chem. Soc. 1983, 105, 5095-5099
**3**	ROO + RH -> R + ROOH	K = 41	Howard, J. A.; Ingold, K. U. Can. J. Chem. 1965, 43, 2729
**4**	ROO + ROO -> P	K = 2.1e7	Howard, J. A.; Ingold, K. U. Can. J. Chem. 1965, 43, 2729

**5**	O2g = O2	k1 = 1.9e+6<br>k2 = 1e+009	assumed. Equilibrium constant from O2 solubility in benzene.
**6**	ROO -> HOO	k = fit	[a]
**7**	QH2 + ROO -> QH + HOOH	fit	
**8**	QH + ROO -> Q + ROOH	k = 2e+009	assumed
**9**	QH+QH->Q+QH2	k = 8e5	Tumanskii, B. L.; Solodovnikov, S. P.; Prokof'ev, A. I.; Bubnov, N. N.; Kabachnik, M. I.; Ivz. Akad. Nauk SSSR Ser. Khim. 1977, 1309 [from Landolt–Börnstein]
**10**	Q+HOO=QH+O2	k1 = fit<br>k2 = fit	
**11**	HOO + RH -> R + HOOH	k = 41	assumed equal to the reaction of ROO•
**12**	ROO + HOO -> O2 + ROOH	k = 6.3000e+008	assumed equal to the reaction HOO• + HOO•
**13**	HOO + HOO -> O2 + HOOH	k = 6.3000e+008	Howard, J. A.; Ingold, K. U. Can. J. Chem. 1965, 43, 2729–2736
**14**	QH2 + HOO -> QH + HOOH	k = 1e6	assumed equal to the reaction ROO• + QH2
**15**	QH + HOO -> Q + HOOH	k = 2e9	assumed
**16**	QH + HOO -> QH2 + O2	k = 2e9	assumed
**17**	QH + HOO -> P	1= 1e8	E. T. Denisov, I. V. Khudyakov Chem. Rev. 1987, 87, 1313-1357

a) the formation of HOO• radicals during styrene autoxidation has been recently demonstrated by Pratt and coworkers (reference 33). This reaction explains the slight retardation of styrene autoxidation by ortho-quinones.

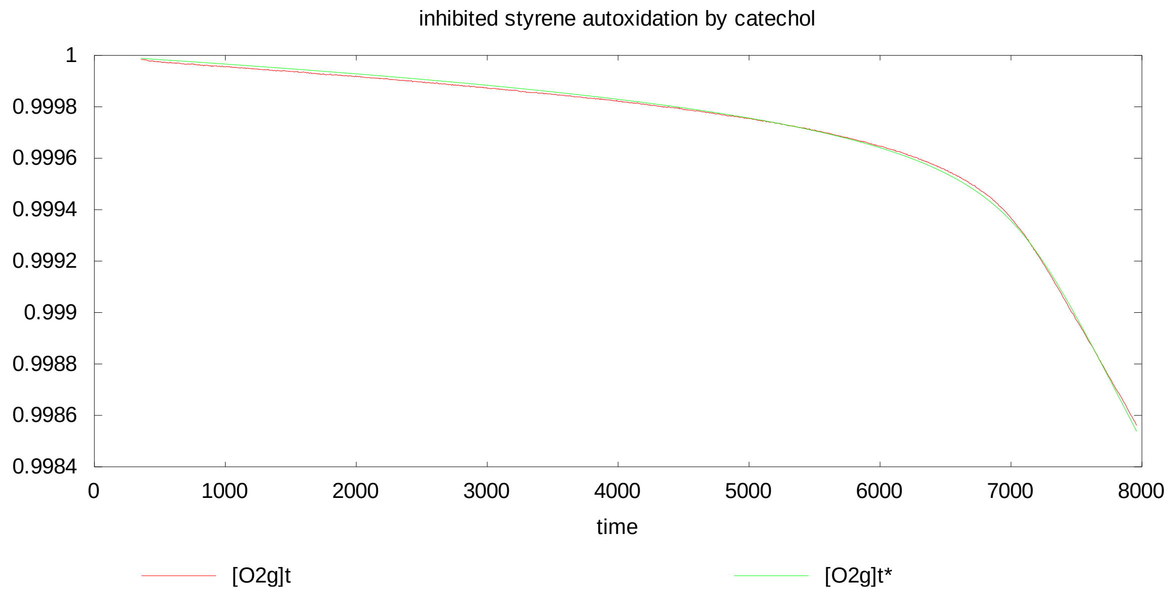

Figure S4. Example of fitting of the autoxidation of styrene inhibited by **1H$_2$**.

Product analysis of CHD autoxidation initiated by AIBN analyzed by GC-MS

Materials: Trimethylsilyl-N,N-dimethylcarbamate (TMSDMC, 98%) was purchased from SantaCruzBiotechnology (Dallas, TX, USA)

Derivatization: $\mathbf{1H_2}$ produced from **1** via autoxidation of CHD in acetonitrile (MeCN) was derivatized by silylating agent, trimethylsilyl-N,N-dimethylcarbamate (TMSDMC). Derivatization reaction of $\mathbf{1H_2}$ was carried out under standard conditions for 2 hours and TMSDMC was added in large excess to assure 100% conversion of $\mathbf{1H_2}$ formed to its silylated derivatives.

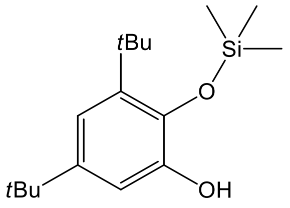

1H(SiMe$_3$)

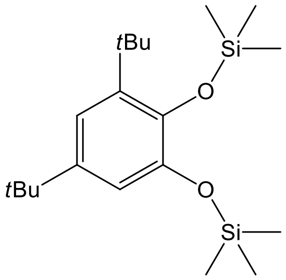

1(SiMe$_3$)$_2$

Structures of the silylated derivatives $\mathbf{1H_2}$,

GC-MS analysis: GC-MS analysis was performed with HP5 capillary column (0.32 mm diameter, 30 m length, 0.25 μm thickness, Hewlett-Packard) with GC-17A Ver.3 Shimadzu and GCMS-QP5050A Shimadzu mass detector. Mass spectrum was obtained by electron impact ionization mode, scanning from 40 m/z to 650 m/z. Inlet temperature was 280C and oven temperature program was as follows: an initial step starts at 60°C; raising at a rate of 10°C/min to 260°C; then holding at 260°C for 5 min.

GC-FID analysis: GC-FID analysis was performed with HP5 capillary column (0.32 mm diameter, 30 m length, 0.25 μm thickness, Hewlett-Packard) with Agilent Technologies 7820A GC System. Inlet temperature and oven temperature program were the same as in the case of GC-MS analysis.

Results: One microliter of the reaction mixture was injected through the inlet of GC-MS or GC-FID with split ratio of 2:1. Two significant molecules, $\mathbf{1HSiMe_3}$ (Mw = 294 Da) and $\mathbf{1(SiMe_3)_2}$ (Mw = 366 Da) were found. Retention time (RT) of the molecules were 10.75 min and 12.25 min respectively. Both molecules were well separated and believed, basing on structural characteristics and mass profiles, to be re-structured products during TMS derivatization originated from hydrogen atom of hydroxyl groups of $\mathbf{1H_2}$

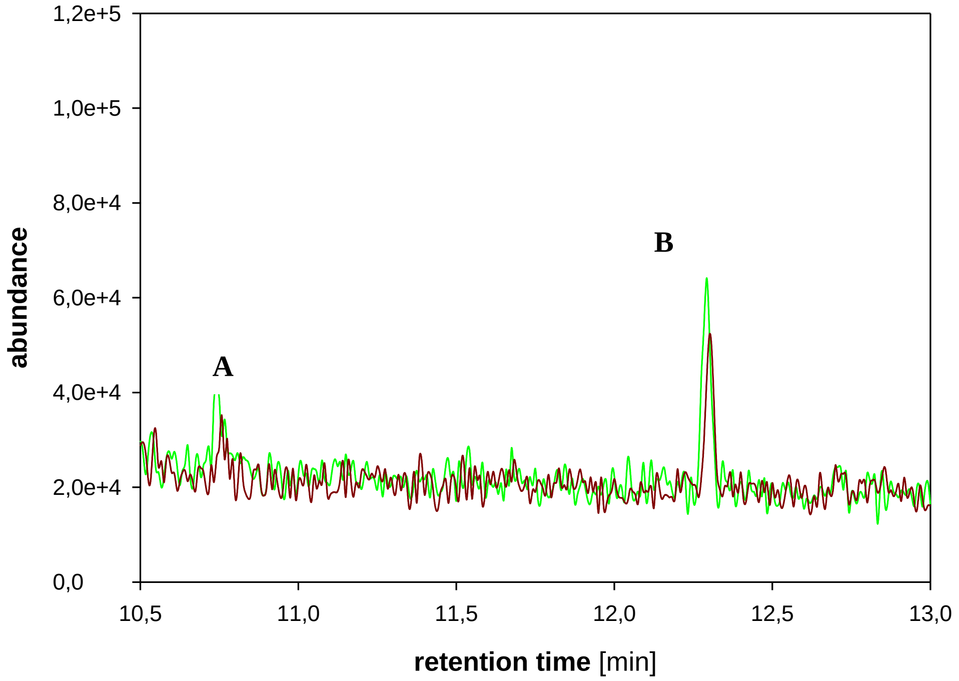

Figure S5. Total ion chromatograms (TIC) of derivatization products (A - **1HSiMe$_3$**; B - **1(SiMe$_3$)$_2$**) of **1H$_2$** formed during autoxidation of CHD (0.13 M) initiated by AIBN (0.05 M) in the presence of **1** (6×10^{-5} M) in MeCN at 30°C (brown line) and reference **1H$_2$** (2×10^{-5} M) in MeCN (green line).

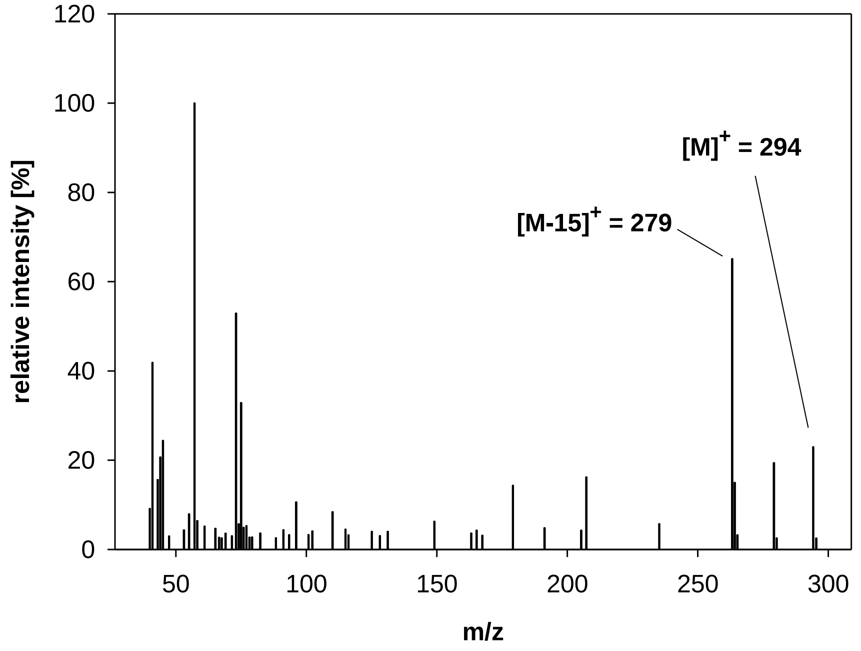

Figure. S6. Mass pattern of compound **1HSiMe$_3$** (Mw = 294 Da).

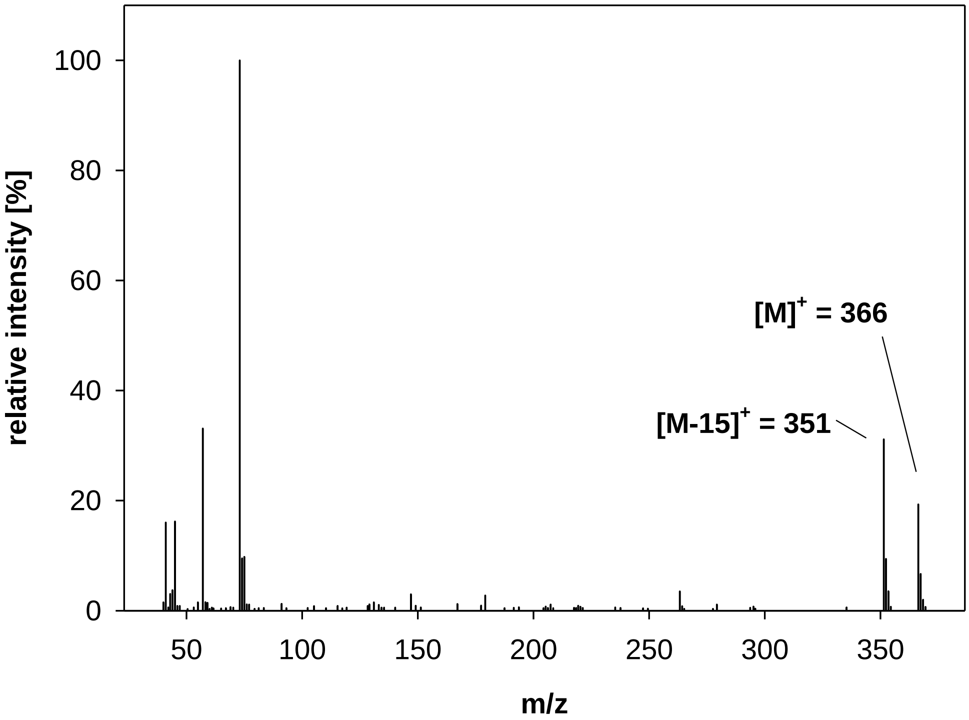

Figure S7. Mass pattern of compound **1(SiMe$_3$)$_2$** (Mw = 366 Da).

GC-FID analysis indicated that during the autoxidation of CHD (0.13 M) in MeCN at 30°C initiated by AIBN (0.05 M) and inhibited by **1** (6×10^{-5} M), the conversion of compound **1** to **1H$_2$** reached the values of 56%, 77% and 80% after 4, 6 and 17 hours, respectively.

Autoxidation in MECN

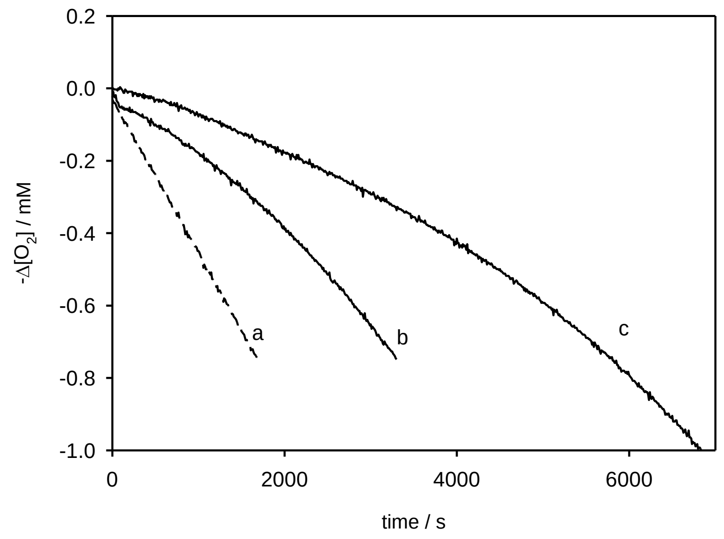

Figure S8. O_2 consumption measured during the autoxidation of CHD (0.13 M) in MeCN initiated by AIBN (0.5 mM) at 30 °C in the absence of antioxidants (a) or in the presence of **1** (a) 5 μM, (b) 25 μM.

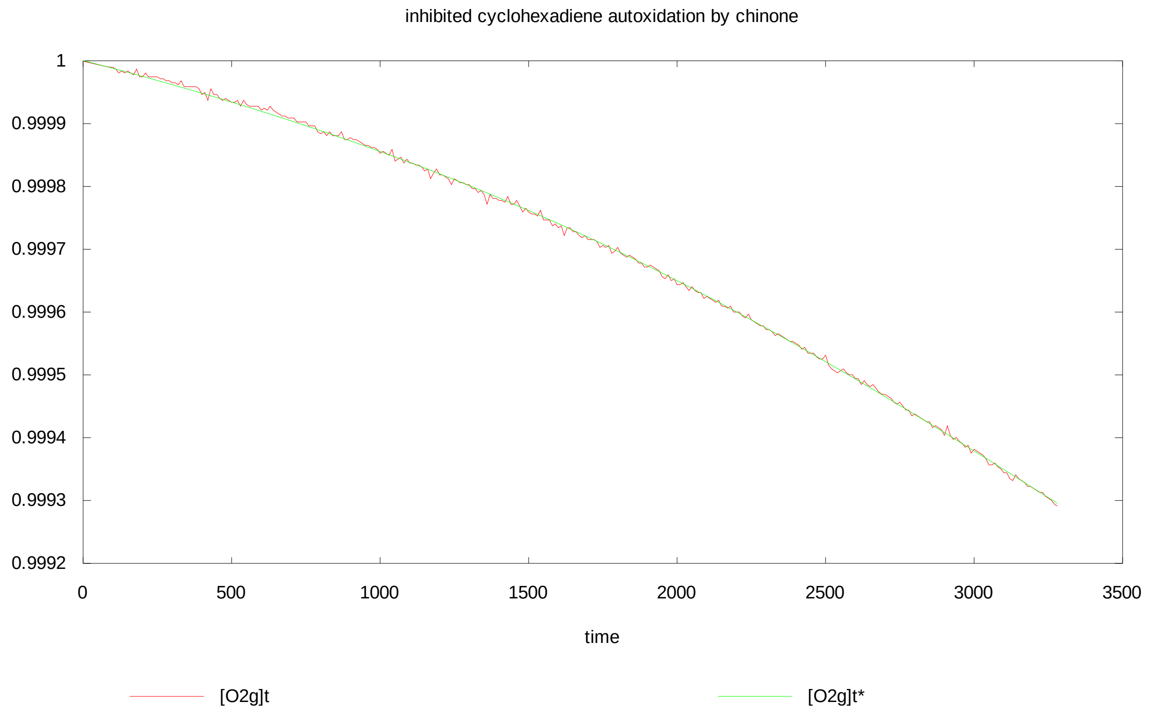

Figure S9. Example of numerical fitting by Copasi of the O_2 consumption trace (b) reported in Figure S8.

Characterization of PDNPs

DLS: the determination of the nanoparticles hydrodynamic diameter distributions was carried out through Dynamic Light Scattering measurements employing a Malvern Nano ZS instrument with a 633 nm laser diode. Samples were housed in disposable polystirene cuvettes of 1 cm optical path length, using water as solvent. The width of DLS hydrodynamic diameter distribuition is indicated by PdI (Polydispersion Index). In case of a mono-modal distribuition (gaussian) calculated by means of cumulant analysis, PdI=$(\sigma/Z_{avg})^2$, where σ is the width of the distribuition and Z_{avg} is average diameter of the particle’s population respectively.

			Size (d.nm):	% Intensity:	St Dev (d.nm):
Z-Average (d.nm):	93,37	Peak 1:	98,46	100,0	23,69
PdI:	0,037	Peak 2:	0,000	0,0	0,000
Intercept:	0,933	Peak 3:	0,000	0,0	0,000
Result quality :	Good				

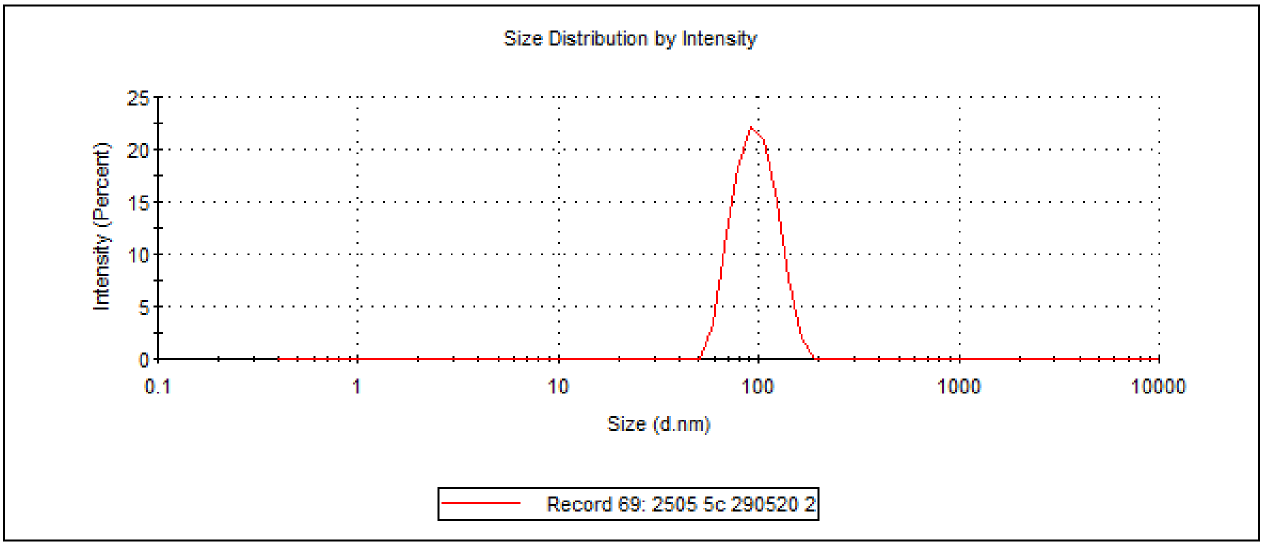

Figure S10. Dynamic Light Scattering measurement of PDA nanoparticles; size distribution by intensity.

TEM Experiments: A Philips CM 100 transmission electron microscope operating at 80 kV was used. For TEM investigations a Formvar foil supported on conventional copper microgrids (400 mesh) was dried up under vacuum after deposition of a drop of nanoparticles solution diluted with water (1:10).

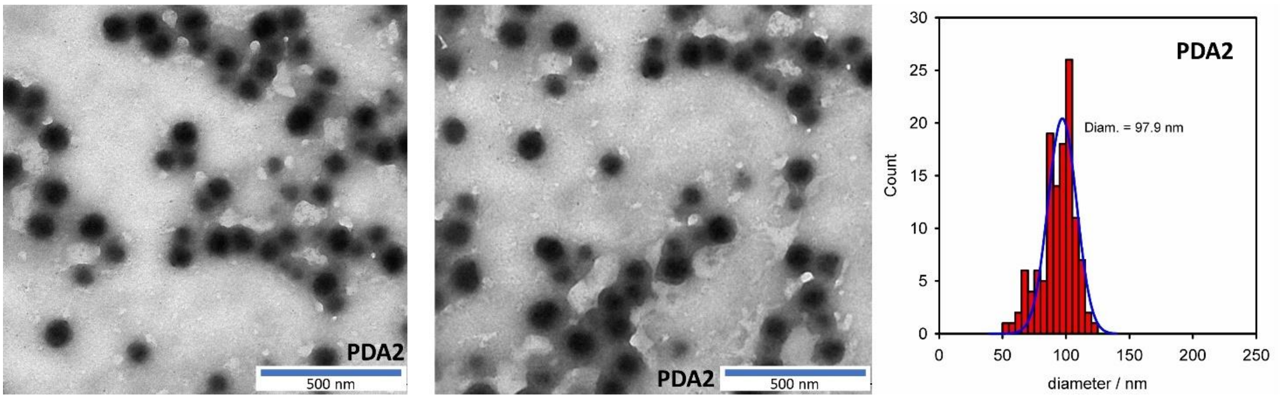

Figure S11. TEM images of PDA nanoparticles.

A

B

C

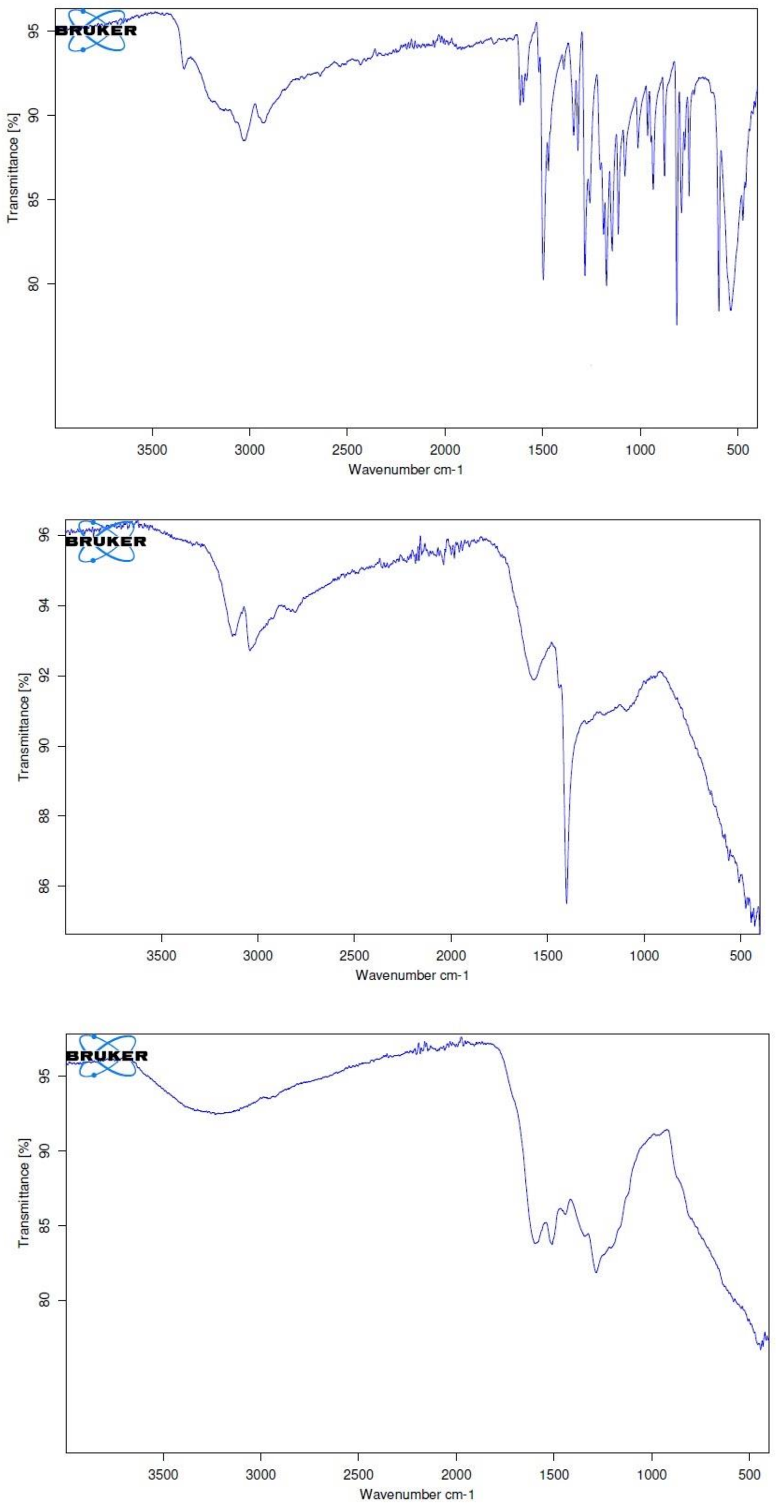

Figure S12. ATR-FT-IR spectra of: (A) Dopamine HCl (solid); (B) non-purified polydopamine nanoparticles; (C) Purified polydopamine nanoparticles.

Inhibition of the autoxidation of styrene/CHD by PDA

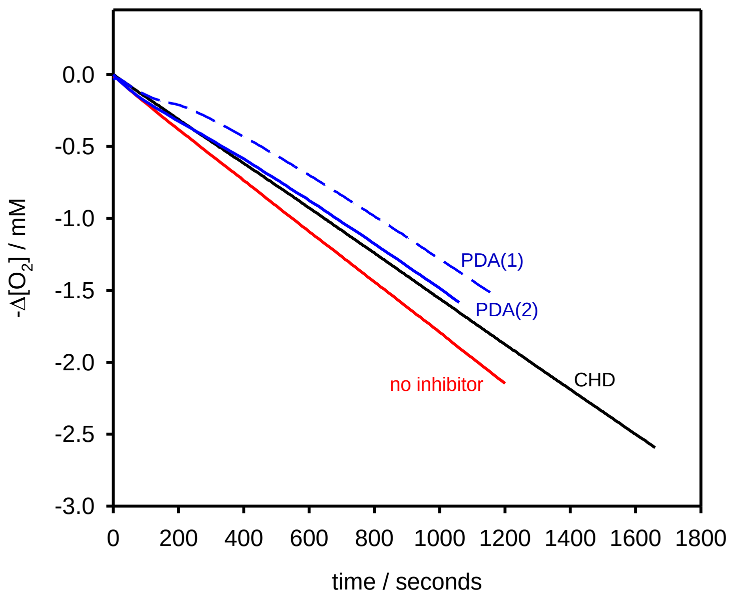

Figure S13. O_2 consumption recorded during the styrene (25%, 2.1 M) autoxidation initiated by AIBN (25 mM) in MeCN without inhibitors (red) and in the presence of: CHD (23 mM), black line; PDA batch 1 (25 μg/mL), blue dashed line; PDA batch 2 (25 μg/mL), blue line.

Results of theoretical calculations

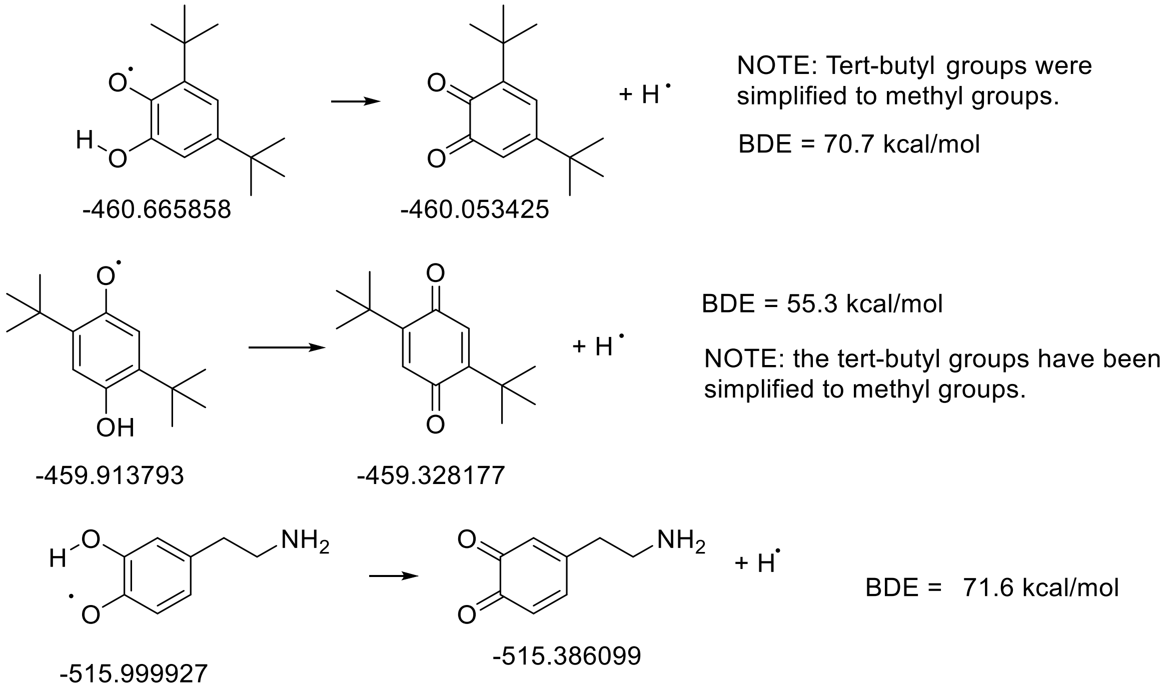

Figure S14. Enthalpy-corrected energies calculated at the CBS-QB3 level, gas phase. ΔH(H•) = -0.497457

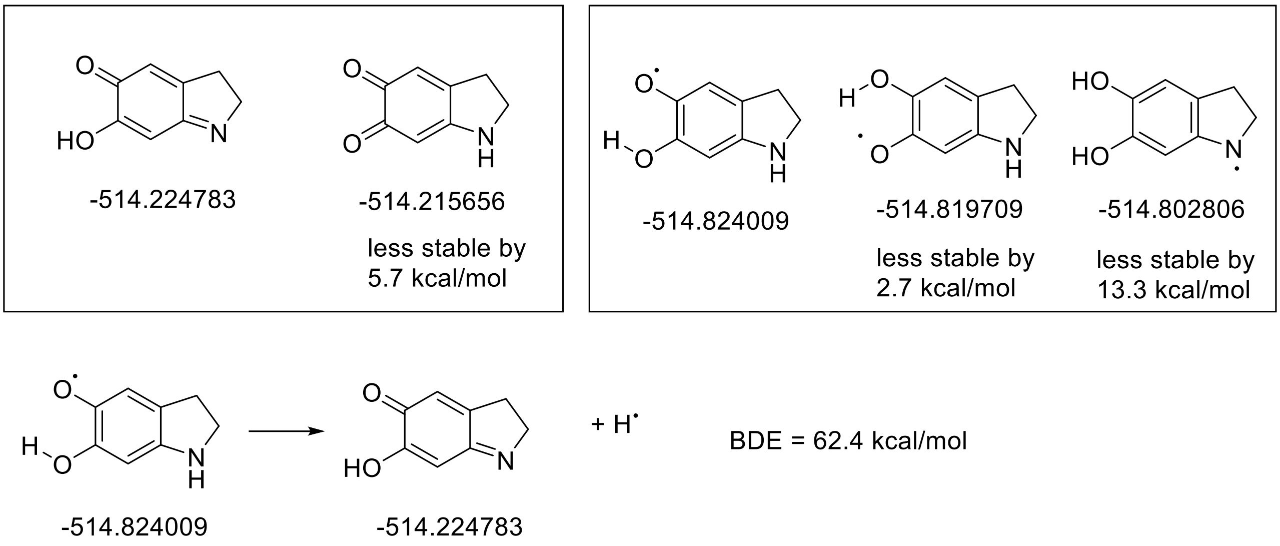

Figure S15. Enthalpy-corrected energies calculated at the CBS-QB3 level, gas phase. ΔH(H•) = -0.497457

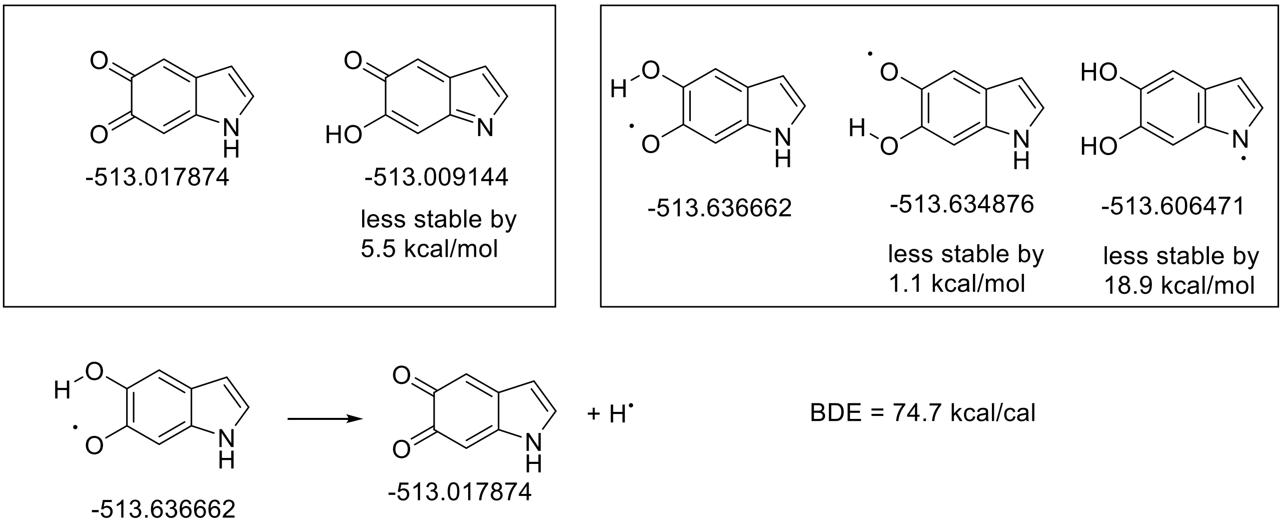

Figure S16. Enthalpy-corrected energies calculated at the CBS-QB3 level, gas phase. ΔH(H•) = -0.497457

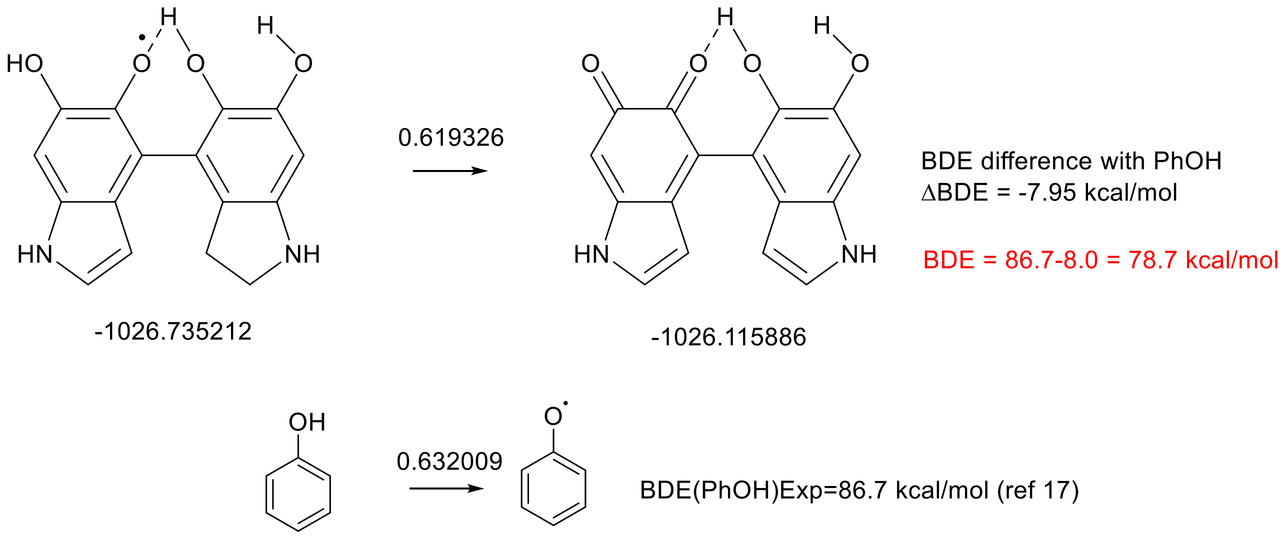

Figure S17. Enthalpy-corrected energies calculated at the B3LYP/6-311++g(d,p) level, gas phase and calculation of the BDE by the isodesmic approach.[9]

References

[1] Y. Huang, Y. Li, Z. Hu, X. Yue, M. T. Proetto, Y. Jones, N. C. Gianneschi, *ACS Cent. Sci.* **2017**, *3*, 564−5699.

[2] X. Jiang, Y. Wang, M. Li, *Sci. Rep.* **2015**, *4*, 6070.

[3] G. W. Burton, K. U. Ingold, *J. Am. Chem. Soc.* **1981**, *103*, 472-6477. M. Lucarini, G.F. Pedulli, L. Valgimigli, R. Amorati, F. Minisci, F, *J. Org. Chem.* **2001**, *66*, 5456-5462

[4] R.; Amorati, G. F.; Pedulli, L. Valgimigli, *Org. Biomol. Chem.* **2011**, *9*, 3792–3800.

[5] R. Amorati, A. Baschieri, L. Valgimigli, *J. Chem.* **2017**, *2017*, 1-12.

[6] S. Hoops, S. Sahle, R. Gauges, C. Lee, J. Pahle, N. Simus, M. Singhal, L. Xu, P. Mendes, U. Kummer, *Bioinformatics* **2006**, *22*, 3067−3074.

[7] J. A. Montgomery, M. J. Frisch Jr, J. W. Ochterski and G. A. Petersson, *J. Chem. Phys.*, **1999**, *110*, 2822

[8] Gaussian 09, Revision D.01, M. J. Frisch, G. W. Trucks, H. B. Schlegel, G. E. Scuseria, M. A. Robb, J. R. Cheeseman, G. Scalmani, V. Barone, G. A. Petersson, H. Nakatsuji, X. Li, M. Caricato, A. Marenich, J. Bloino, B. G. Janesko, R. Gomperts, B. Mennucci, H. P. Hratchian, J. V. Ortiz, A. F. Izmaylov, J. L. Sonnenberg, D. Williams-Young, F. Ding, F. Lipparini, F. Egidi, J. Goings, B. Peng, A. Petrone, T. Henderson, D. Ranasinghe, V. G. Zakrzewski, J. Gao, N. Rega, G. Zheng, W. Liang, M. Hada, M. Ehara, K. Toyota, R. Fukuda, J. Hasegawa, M. Ishida, T. Nakajima, Y. Honda, O. Kitao, H. Nakai, T. Vreven, K. Throssell, J. A. Montgomery, Jr., J. E. Peralta, F. Ogliaro, M. Bearpark, J. J. Heyd, E. Brothers, K. N. Kudin, V. N. Staroverov, T. Keith, R. Kobayashi, J. Normand, K. Raghavachari, A. Rendell, J. C. Burant, S. S. Iyengar, J. Tomasi, M. Cossi, J. M. Millam, M. Klene, C. Adamo, R. Cammi, J. W. Ochterski, R. L. Martin, K. Morokuma, O. Farkas, J. B. Foresman, and D. J. Fox, Gaussian, Inc., Wallingford CT, 2016.

[9] M. Guerra, R. Amorati, G. F. Pedulli *J. Org. Chem.* **2004**, *69*, 5460-5467

[10] M. Griesser, R. Shah, A. T. V. Kessel, O. Zilka, E. A. Haidasz, D. A. Pratt, *J. Am. Chem. Soc.* **2018**, *140*, 3798−3808.

[11] R. Amorati, P. T. Lynett, L. Valgimigli, D. A. Pratt, *Chem.-Eur. J.* **2012**, *18*, 6370 – 6379.

[12] J. A. Howard, K. U. Ingold, *Can. J. Chem.* **1967**, *45*, 785–792.

[13] R. Amorati, R, L. Valgimigli, P. Diner, K. Bakhtiari, M. Saeedi, L. Engman, *Chem.-Eur. J.* **2013**, *19*, 7510-7522.

[14] E. T. Denisov, I. V. Khudyakov, *Chem. Rev.* **1987**, *87*, 1313-1357.

[15] A. Baschieri, R. Amorati, L. Valgimigl, L. Sambri, *J. Org. Chem.* **2019**, *84*, *21*, 13655-13664.

[16] R. Amorati, A. Baschieri, G. Morroni, R. Gambino, L. Valgimigli, *Chem.- Eur. J.* **2016**, *22,* 7924-7934.

[17] P. Mulder, H.-G. Korth, D. A. Pratt, G. A. DiLabio, L. Valgimigli, G. F. Pedulli, K. U. Ingold *J. Phys. Chem. A* **2005**, *109,* 2647-2655.
